# Correlation between electrode location and clinical efficacy of deep brain stimulation of the globus pallidus internus in isolated generalized dystonia

**DOI:** 10.3389/fneur.2026.1779301

**Published:** 2026-05-28

**Authors:** Jingchao Wu, Guanyu Zhu, Jianguo Zhang

**Affiliations:** 1 Department of Neurosurgery, Tianjin Huanhu Hospital, Tianjin, China; 2 Tianjin Key Laboratory of Cerebral Blood Flow Reconstruction and Head and Neck Tumor New Technology Translation, Tianjin, China; 3 Department of Neurosurgery, Beijing Tiantan Hospital, Capital Medical University, Beijing, China

**Keywords:** DBS (deep brain stimulation), dystonia, GPi (globus pallidus internus), isolated generalized dystonia, VTA (volume of tissue activated)

## Abstract

**Objectives:**

This study aimed to investigate the correlation between electrode location, volume of tissue activated (VTA), and clinical outcomes in patients with isolated generalized dystonia (IGD) undergoing deep brain stimulation (DBS) of the globus pallidus internus (GPi), and to identify predictors of treatment efficacy.

**Methods:**

A total of 17 IGD patients who received bilateral GPi-DBS at Beijing Tiantan Hospital from January 2016 to December 2021 and had complete follow-up data were enrolled. The follow-up duration ranged from 12 to 60 months. Preoperative 3.0T magnetic resonance imaging (MRI) and postoperative computed tomography (CT) were used for electrode localization. Clinical efficacy was evaluated using the Burke–Fahn–Marsden dystonia rating scale (BFMDRS), including the motor subscale (BFMDRS-M) and disability subscale (BFMDRS-D). Additionally, the Hamilton anxiety scale (HAMA), Hamilton depression scale (HAMD), montreal cognitive assessment (MOCA), and mini-mental state examination (MMSE) were used to assess anxiety, depression, and cognitive function. Demographic, clinical, psychological, and cognitive variables were included in statistical analyses.

**Results:**

Results showed that postoperative BFMDRS-M and BFMDRS-D scores were significantly lower than preoperative scores (both *P* < 0.001), while no significant changes were observed in HAMA, HAMD, MOCA, or MMSE scores. In the Montreal Neurological Institute (MNI) space, the *Z*-axis coordinates of bilateral active electrode contacts were significantly correlated with the improvement rate of BFMDRS-M (left side: *P* = 0.004; right side: *P* = 0.041), with electrode positions closer to the ventral GPi associated with better therapeutic effects. No significant correlation was found between the VTA of the entire GPi or its subregions and BFMDRS-M improvement rate (all *P* > 0.05). Multivariate linear regression analysis identified preoperative HAMA score as an independent predictor of poor motor outcomes (*P* = 0.0189).

**Conclusions:**

GPi-DBS significantly improves motor function in IGD patients. Precise electrode targeting of the ventral GPi and preoperative assessment and intervention for anxiety symptoms are crucial for optimizing DBS treatment outcomes. This study provides clinical evidence for target selection and efficacy prediction in DBS treatment for IGD patients.

## Introduction

Dystonia is a movement disorder characterized primarily by abnormal movements or postures caused by sustained or intermittent muscle contractions (1). Isolated generalized dystonia (IGD) must simultaneously meet the diagnostic criteria for both isolated dystonia and generalized dystonia. This condition severely impacts patients' quality of life and poses growing clinical challenges. Globus pallidus internus (GPi)-Deep brain stimulation (DBS) is an important treatment method for refractory dystonia (2–4). In most clinical studies, the severity of dystonia in patients with primary dystonia generally improved by 50%−60% (5–7). However, the results are often variable, with randomized controlled trial results showing up to 25% non-responders (improvement rate < 25%), even in carefully selected groups of primary dystonia patients (5).

Although DBS of the GPi is well established for generalized dystonia, most previous studies have enrolled heterogeneous populations, including mixed subtypes and etiologies. It remains unclear whether their conclusions can be directly applied to patients with isolated generalized dystonia as a distinct clinical group (8). Inconsistencies also exist regarding which GPi subregions are most critical for therapeutic benefit, especially within homogeneous cohorts (9). At present, the factors influencing the efficacy of DBS in the treatment of IGD remain poorly understood at present (10, 11). This study aims to investigate the correlation between electrode location, VTA, and clinical outcomes in IGD patients receiving DBS in the GPi. Additionally, we incorporate disease duration, age at surgery, age at onset, baseline BFMDRS-M scores, and scores from Hamilton anxiety rating scale (HAMA), Hamilton depression rating scale (HAMD), mini-mental state examination (MMSE), and Montreal cognitive assessment (MOCA) into a linear regression model to aid in patient selection and outcome prediction.

## Materials and methods

### Patients

This study included patients with IGD who underwent bilateral GPi-DBS treatment in the Department of Functional Neurosurgery, Beijing Tiantan Hospital, Capital Medical University, from January 2016 to December 2021, and had complete follow-up data. The follow-up duration ranged from 12 to 60 months. Inclusion criteria: (1) Diagnosis of IGD confirmed by movement disorder specialists; (2) Preoperative MRI showing no structural abnormalities (e.g., tumors, hemorrhage); (3) No history of severe psychiatric/cognitive disorders, epilepsy, thalamotomy, or other neurosurgical procedures affecting neural function; (4) Age ≥ 16 years at the time of cognitive/psychological assessment. Exclusion criteria: (1) Secondary dystonia (e.g., post-stroke, neurodegenerative disease); (2) MRI evidence of brain structural lesions; (3) Severe psychiatric disorders (e.g., schizophrenia) or cognitive impairment (MOCA < 10). A total of 17 IGD patients were enrolled. The study was conducted in accordance with the Declaration of Helsinki and approved by the Ethics Committee of Beijing Tiantan Hospital (Ethics No: KY2022-006-02). Written informed consent was obtained from all patients or their guardians.

### Imaging, surgical and programming procedures

All patients underwent 3.0T magnetic resonance imaging (MRI; MAGNETOM Prisma, Siemens, Germany) 1 day preoperatively, including 3d T1 and T2 navigation scans for high-resolution brain structural imaging to support target localization and surgical planning. High-resolution 3d T1-weighted images were obtained using Voxel size with 1.0 × 1.0 × 1.0 mm, NEX = 1, and acquisition time of 4 min. High-resolution T2-weighted images were acquired using Voxel size with 1.0 × 1.0 × 2.0 mm, NEX = 1, and acquisition time of 3 min. Surgery was performed under general anesthesia (GA). Bilateral arc incisions were made on the forehead; after hemostasis, skull drilling and dural cauterization were conducted. Guided by the stereotactic frame, catheters and recording electrodes were inserted, and microelectrode recording (MER) confirmed GPi localization before implanting deep brain electrodes (PINS L302, PINS Medical Co., Beijing, China; Medtronic 3387, Medtronic Inc., Minneapolis, MN, USA). Postoperatively, thin-slice head CT was performed to rule out complications (e.g., intracranial hemorrhage). SurgiPlan fused preoperative MRI and postoperative CT to verify electrode position deviation. After 3–7 days of satisfactory external stimulation, a second-stage IPG (implantable pulse generator) implantation was done under GA (PINS G102/G102RZ, PINS Medical Co., Beijing, China; or Activa RC, Medtronic Inc., Minneapolis, MN, USA), with IPG placed under the clavicle.

### Clinical assessments

All patients underwent standardized video recordings for dystonia evaluation. Assessments were performed preoperatively and at the last follow-up (12–60 months post-DBS activation). The Burke–Fahn–Marsden Dystonia Rating Scale (BFMDRS) was used: BFMDRS-M (motor subscale, 0–120 points) evaluates dystonia severity across multiple body regions in both the resting state (*R*) and the provoked action state (*P*), and BFMDRS-D (disability subscale, 0–30 points) assesses functional impairment. Hamilton Anxiety Scale (HAMA): A 14-item scale (0–4 points per item) evaluating anxiety severity. Hamilton Depression Scale (HAMD): a 24-item scale (0–4 points per item) evaluating depression severity. Montreal Cognitive Assessment (MOCA): A 30-item scale assessing multiple cognitive domains. Mini-Mental State Examination (MMSE): A 30-item scale for rapid cognitive screening.

### DBS electrode localization and VTA calculation

Lead-DBS V3.0 software (https://www.lead-dbs.org/) was used for electrode localization, with coordinates converted to MNI and AC-PC standard spaces. VTA was calculated using Lead-DBS V3.0 based on stimulation parameters at the last follow-up (frequency, pulse width, voltage, stimulation mode). Overlap analysis: VTA overlap with GPi was quantified (mm^3^) (12, 13).

### Statistical analysis

All analyses were performed using SPSS 23.0 (IBM Corp., Armonk, NY, USA). Continuous variables were presented as mean ± standard deviation (SD). Paired *t*-tests or Wilcoxon signed-rank tests were used for preoperative-postoperative comparisons. Kruskal–Wallis tests were used for multi-group comparisons. Categorical variables were presented as proportions, with χ^2^ tests for group comparisons. Pearson correlation analysis was used to assess the relationship between BFMDRS improvement rate and VTA (correlation coefficient *R*: >0.5 = strong, 0.3–0.5 = weak, < 0.3 = no correlation).

Clinical variables (disease duration, age at surgery/onset, baseline BFMDRS-M, HAMA, HAMD, MMSE, MOCA) were included in univariate linear regression models. Variables with *P* < 0.05 in univariate analysis were entered into a multivariate linear regression model to identify independent predictors. Statistical significance was set at *P* < 0.05.

## Results

### Clinical data

A total of 17 patients underwent GPi-DBS (10 males, 7 females). The mean age at surgery was 26.12 ± 13.3 years, mean age at onset was 17.12 ± 16.75 years, mean disease duration was 107.18 ± 99.22 months, and mean follow-up duration was 34.5 ± 15.2 months. Stimulation parameters at last follow-up are shown in Table 1.

**Table 1:** Characteristics and stimulation parameters of each patient with generalized isolated dystonia.

Case	Sex	Age at onset (yrs)	Age at Surgery (yrs)	Duration of symptoms (mos)	Gene mutation	Stimulation parameters with best response (contact, pulse width, frequency, amplitude, left/right)
1	F	1	22	252	NA	Case (+) 5 (–) 6 (–)/Case (+) 2 (–) 3 (–), 60/60, 170/170, 3.0/2.8
2	M	1	25	288	No	Case (+)6 (–)/Case (+) 2 (–), 70/70, 140/140, 3.5/3.7
3	M	27	29	16	NA	Case (+) 5 (–) 6 (–)/Case (+) 2 (–) 3 (–), 60/70, 160/160, 3.5/3.0
4	F	40	45	60	NA	Case (+) 6 (–)/Case (+) 1 (–), 60/60, 130/130, 3.4/3.0
5	M	37	40	36	No	Case (+) 6 (–)/Case (+) 2 (–), 60/70, 150/150, 3.5/3.5
6	F	4	9	60	No	Case (+) 6 (–) 7 (–)/Case (+) 1 (–) 2 (–), 90/70, 160/160, 2.9/2.8
7	M	5	29	288	NA	Case (+) 7 (–)/Case (+) 3 (–), 60/60, 130/130, 3.6/3.0
8	M	8	12	48	NA	Case (+) 6 (–) 7 (–)/Case (+) 1 (–) 2 (–), 100/70, 145/145, 2.95/2.85
9	M	9	10	6	TOR1A	Case (+) 5 (–)/Case (+) 1 (–), 70/80, 150/150, 2.0/2.75
10	F	36	38	24	NA	Case (+) 5 (–) 6 (–)/Case (+) 1 (–) 2 (–), 70/80, 140/140, 2.7/2.9
11	M	6	14	96	TOR1A	Case (+) 5 (–)/Case (+) 1 (–), 90/90, 160/160, 2.5/3.0
12	M	8	12	48	No	Case (+) 6 (–) 7 (–) 8 (–)/Case (+) 2 (–) 4 (–), 60/100, 130/130, 3.5/3.5
13	M	7	24	204	GNAL	Case (+) 5 (–)/Case (+) 2 (–), 90/90, 170/170, 3.4/3.2
14	M	8	21	156	TOR1A	Case (+) 5 (–) 6 (–)/Case (+) 1 (–) 2 (–), 80/80, 160/160, 2.4/2.8
15	F	43	45	24	NA	Case (+) 5 (–)/Case (+) 2 (–), 100/70, 140/140, 3.4/3.5
16	F	47	50	36	NA	Case (+) 6 (–)/Case (+) 2 (–), 90/90, 150/150, 3.4/3.2
17	F	4	19	180	No	Case (+) 9 (–)/Case (+) 1 (–), 60/60, 150/150, 2.6/2.7

F, female; M, male; none, no gene mutation; NA, not available (patient refused genetic testing).

### Clinical efficacy of GPi-DBS

Postoperative BFMDRS-M and BFMDRS-D scores were significantly lower than preoperative scores. The mean BFMDRS-M score decreased from 47.09 ± 23.68 preoperatively to 27.68 ± 25.24 at last follow-up (*P* = 0.0002). The mean BFMDRS-D score decreased from 16.18 ± 5.74 preoperatively to 9.71 ± 7.49 at last follow-up (*P* = 0.0009). There were no significant improvements in HAMA or HAMD scores. The mean HAMA score decreased from 16.00 ± 8.46 preoperatively to 14.50 ± 8.58 at last follow-up (*P* = 0.101). The mean HAMD score decreased from 15.83 ± 7.85 preoperatively to 14.83 ± 8.24 at last follow-up (*P* = 0.067). MOCA and MMSE scores showed no significant changes. The mean MOCA score increased from 20.83 ± 3.97 preoperatively to 21.25 ± 4.41 at last follow-up (*P* = 0.516). The mean MMSE score decreased slightly from 22.16 ± 4.41 preoperatively to 21.92 ± 4.25 at last follow-up (*P* = 0.714).

### The active contact locations in MNI/AC-PC and its relationship with the improvement rate of motor symptoms in IGD patients

MNI Space: the mean coordinates of left-sided activation contacts were: *X* = −21.37 ± 0.91 mm, *Y* = −7.09 ± 1.71 mm, *Z* = −5.07 ± 1.94 mm. The mean coordinates of right-sided contacts were: *X* = 21.03 ± 0.97 mm, *Y* = −6.56 ± 1.01 mm, *Z* = −5.08 ± 1.70 mm. AC-PC Space: The mean coordinates of left-sided activation contacts were: *X* = −20.12 ± 1.04 mm, *Y* = 4.22 ± 1.23 mm, *Z* = −1.22 ± 2.12 mm. The mean coordinates of right-sided contacts were: *X* = 19.53 ± 1.13 mm, *Y* = 5.47 ± 0.78 mm, *Z* = −1.09 ± 2.01 mm.

According to the improvement rate of the Burke–Fahn–Marsden Dystonia Rating Scale-Motor (BFMDRS-M), patients were divided into responders (improvement rate > 50%), intermediate responders (improvement rate 25%−50%), and non-responders (improvement rate < 25%) (14). In the Montreal Neurological Institute (MNI) space, there were statistically significant differences in the bilateral *Z*-axis coordinate values among the three groups (left side: *P* = 0.004; right side: *P* = 0.041), while no statistically significant differences were observed in the bilateral *X*-axis and *Y*-axis coordinate values (*P* > 0.05, Table 2). In the anterior commissure-posterior commissure (AC-PC) space, there were no statistically significant differences in the *X*-axis, *Y*-axis, or *Z*-axis coordinate values among the three groups (*P* > 0.05, Table 2).

**Table 2:** Comparison results of active contact coordinates in MNI and AC-PC space in different BFMDRS-M Improvement rate groups [M (IQR), mm].

Group	Number of cases	Left			Right		
		*X*	*Y*	*Z*	*X*	*Y*	*Z*
MNI space							
Improvement rates < 25% group	7	−21.54 ± 1.14	−6.82 ± 1.78	−3.25 ± 0.98	20.45 ± 1.13	−6.20 ± 1.11	−3.82 ± 1.28
Improvement rates 25%−50% group	1	−22.37	−4.86	−6.16	22.68	−5.37	−4.56
Improvement rates >50% group	9	−21.14 ± 0.68	−7.54 ± 1.61	−6.36 ± 1.38	21.29 ± 0.44	−6.97 ± 0.81	−5.77 ± 1.18
Kruskal–Wallis χ^2^		1.937	2.423	11.016	3.765	3.087	6.835
*P* value		0.380	0.298	**0.004**	0.392	0.152	**0.033**
AC-PC space							
Improvement rates < 25% group	7	−20.65 ± 0.83	4.40 ± 1.33	−1.07 ± 2.15	19.02 ± 1.22	5.27 ± 0.85	−0.94 ± 2.38
Improvement rates 25%−50% group	1	−19.96	5.39	1.45	20.05	5.83	2.17
Improvement rates >50% group	9	−19.91 ± 1.16	3.94 ± 1.89	−1.63 ± 2.11	19.87 ± 1.01	5.56 ± 0.79	−1.56 ± 1.49
Kruskal–Wallis *χ^2^*		2.154	1.937	2.689	1.310	0.997	2.297
*P* Value		0.341	0.380	0.261	0.520	0.607	0.317

*P* values were calculated using Kruskal–Wallis tests. Bold values indicate statistically significant differences.

### Relationship between GPi/subregion VTA and BFMDRS improvement rate

**Figure 1:**
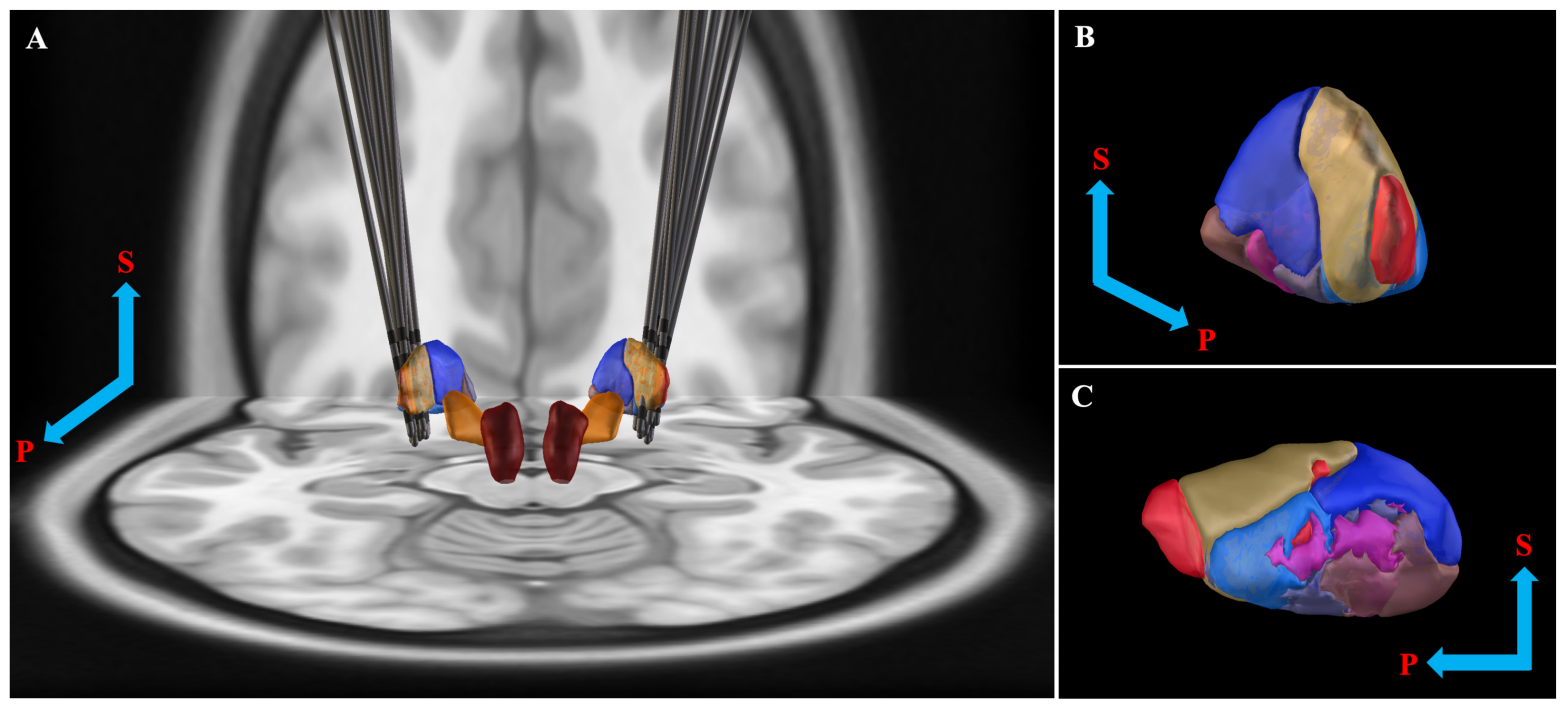
**(A)** 3D illustration of all active electrode contacts: Electrode position of 17 IGD patients (Yellow nucleus: STN. Red nucleus: red nucleus. multi-colored nucleus: GPi). **(B, C)** GPi and subregions: GPi sensory subregions (Magent). GPi primary motor subregions (Red). GPi premotor subregions (Yellow). GPi postparietal subregions (Light Blue). GPi occipital subregions (Slate Blue). GPi temporal subregions (Brown). GPi prefrontal subregions (Deep Blue). *P*, posterior; *S*, superior.

GPi was subdivided into subregions based on efferent pathways: sensory, primary motor, premotor, posterior parietal, occipital, temporal, and prefrontal regions using the DISTAL atlas (15), which provides topographically defined subregions of the globus pallidus based on anatomical connectivity profiles. The sensorimotor region (sensory + primary motor + premotor regions) is considered the optimal target for GPi-DBS in dystonia (Figure 1).

Mean VTA volumes of GPi subregions were: sensory (12.34 ± 8.31 mm^3^), primary motor (79.52 ± 32.15 mm^3^), premotor (70.72 ± 33.85 mm^3^), posterior parietal (63.74 ± 25.04 mm^3^), occipital (5.25 ± 7.29 mm^3^), temporal (1.20 ± 3.18 mm^3^), prefrontal (10.93 ± 19.31 mm^3^), sensorimotor (115.69 ± 51.41 mm^3^), and total GPi (196.95 ± 84.10 mm^3^). Pearson correlation analysis showed no significant correlation between BFMDRS-M improvement rate and VTA of GPi or its subregions (all *P* > 0.05; Table 3).

**Table 3:** The correlation between VTA and the BFMDRS Improvement RATE.

VTA	BFMDRS-M improvement rate	
	*R*	*P*
GPi sensory	−0.142	0.586
GPi primary motor	−0.126	0.630
GPi premotor	0.016	0.952
GPi postparietal	0.193	0.459
GPi occipital	−0.141	0.590
GPi temporal	−0.209	0.420
GPi prefrontal	−0.308	0.228
GPi sensorimotor	−0.122	0.642
GPi	−0.109	0.677

GPi sensorimotor = GPi sensory + GPi primary motor + GPi premotor; *R*, correlation coefficients; *P, p* value; *P* and *R* were generated from Pearson correlation analysis.

### Predictors of motor outcomes

**Figure 2:**
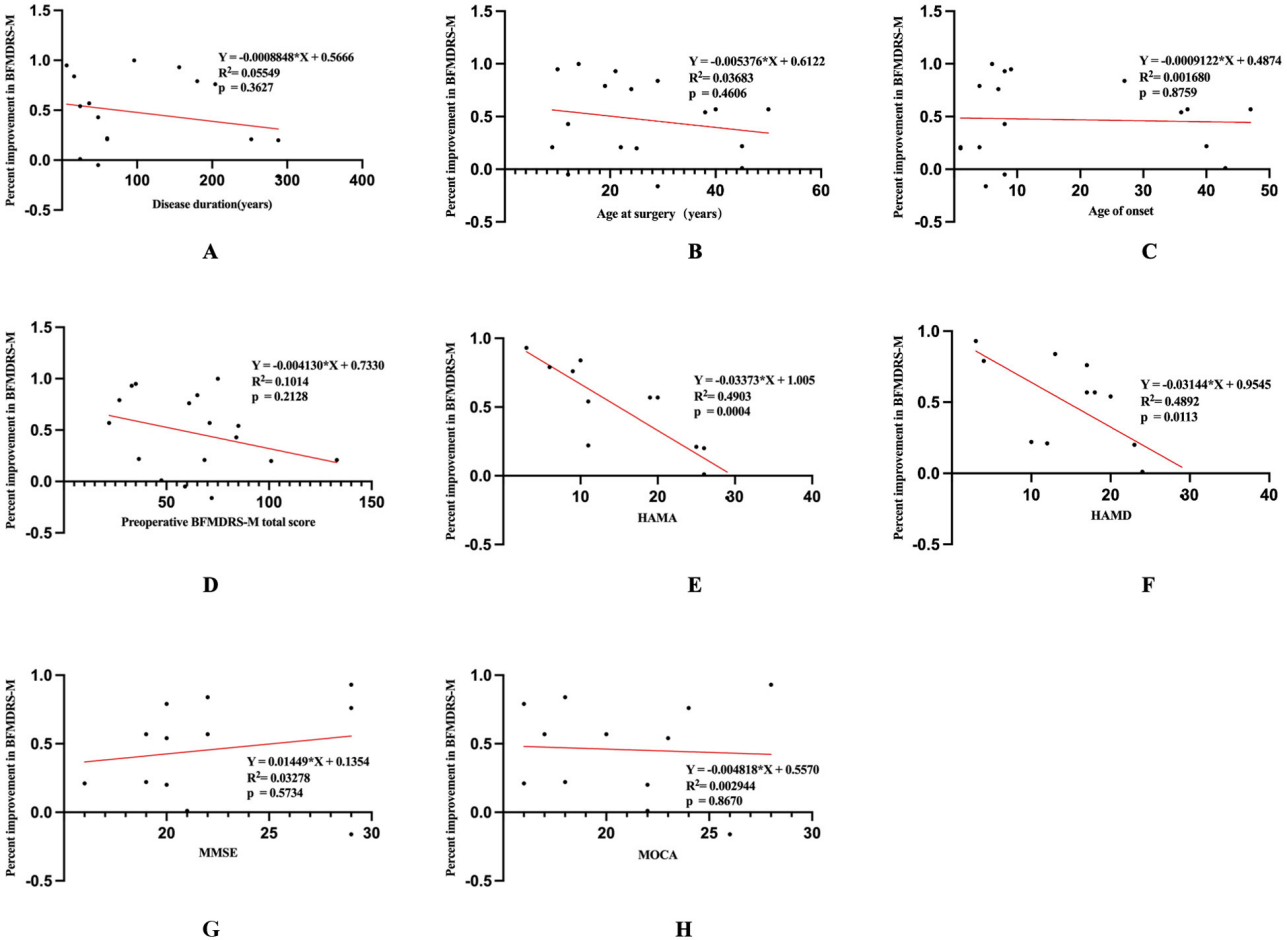
Predictors of motor outcomes after GPi-DBS. A simple linear regression model was constructed to assess candidate predictive factors for movement outcomes, including baseline variables (demographic and clinical). Significant negative correlations were observed between motor outcome and preoperative HAMA **(E)**, preoperative HAMD **(F)**, while Disease duration **(A)**, Age at surgery **(B)**, Age of onset **(C)**, Preoperative BFMDRS-M total score **(D)**, MMSE score (**G)** and MOCAscore **(H)** showed no significant correlations.

Univariate linear regression included variables: disease duration, age at surgery, age at onset, Preoperative BFMDRS-M, HAMA, HAMD, MMSE, MOCA (Figure 2). Variables with *P* < 0.05 (HAMA: *P* = 0.0004; HAMD: *P* = 0.0113) were entered into the multivariate model. The final multivariate linear regression model (adjusted *R*^2^ = 0.6726, *P* < 0.001) identified preoperative HAMA score as an independent predictor of poor motor outcomes (standardized β = −0.03124, 95% CI: −0.05598 to −0.006496, *P* = 0.0189). HAMD was not an independent predictor (*P* = 0.7776) (Table 4).

**Table 4:** Factors influencing the BFMDRS-M outcome in patients with IGD after bilateral GPi-DBS.

Variable	Univariable analysis			Multivariable analysis		
	β	95% CI	*p*-value	β	95% CI	*p*-value
Disease duration	−0.236	−0.003 to 0.001	0.363			
Age at surgery	−0.192	−0.021 to 0.010	0.461			
Age of onset	−0.041	−0.01315 to 0.01132	0.8759			
BFMDRS-M total score	−0.318	−0.01089 to 0.002635	0.2128			
HAMA	−0.854	−0.04819 to −0.01926	**0.0004**	**−0.031**	−0.05598 to −0.006496	**0.0189**
HAMD	−0.699	−0.05408 to −0.008806	**0.0113**	**−0.004**	−0.03179 to 0.02454	0.7776
MMSE	0.181	−0.04098 to 0.06997	0.5734			
MOCA	−0.054	−0.06729 to 0.05765	0.8670			

Bold values indicate statistically significant differences.

### Adverse events

Postoperatively, no cases of intracranial hemorrhage or infection were detected following DBS implantation. One patient developed focal cervical muscular spasm, which was effectively relieved by reducing stimulation intensity. Another patient experienced accidental deactivation of the stimulator at 4 months post-surgery, with subsequent reactivation. Three patients reported dysarthria, vertigo, and paresthesia; these adverse events were resolved through parameter reprogramming. No dyskinesia was observed in any study participant.

## Discussion

DBS exerts its therapeutic effects on dystonia through complex neural mechanisms. Unlike Parkinson's disease, where DBS provides immediate symptom relief, dystonia patients often show delayed responses (days to weeks), and symptom recurrence may be absent for months after stimulation cessation—suggesting DBS induces neuroplastic changes in dystonic neural networks (16). Dystonia is increasingly recognized as a “network disorder”, and DBS acts as a network-based intervention targeting abnormal activity in the cortico-basal ganglia-thalamo-cerebellar circuit (17).

GPi-DBS is a well-established treatment for drug-refractory focal, segmental, or generalized dystonia, with most studies reporting >50% symptom improvement (18, 19). However, 10%−25% of patients achieve < 25%−30% improvement (6). The causes of this variability remain unclear, but electrode position is a key modifiable factor—previous studies identified improper electrode placement as the most common reason for poor outcomes (20).

Traditional GPi targeting relies on anatomical landmarks (e.g., ventral border of the optic tract) and intraoperative microelectrode recording (21). Tisch et al. (22) reported that more posterior electrode placement along the AC-PC axis correlated with better generalized dystonia improvement, while Cheung et al. (23) identified the middle-posterior 1/3 of GPi as the optimal target for DYT1 dystonia. Our findings align with these studies, showing that ventral GPi contacts (lower *Z* values in MNI space) were associated with better BFMDRS-M improvement. This anatomical specificity is supported by GPi's structure: the ventral GPi corresponds to the sensorimotor region, which serves as a relay for motor fibers (24). Stimulation of this region modulates abnormal motor circuits by inhibiting excessive neural activity in the basal ganglia—reducing muscle spasms and improving motor coordination (25). Additionally, the ventral GPi contains overlapping projections of the ansa lenticularis and lenticular fasciculus, two major efferent pathways of the basal ganglia; stimulating this region allows simultaneous modulation of both pathways, enhancing therapeutic efficacy (26, 27).

A notable discrepancy was observed between significant findings in MNI space and non-significant results in AC-PC space. This may be attributed to several factors, including differences in spatial normalization algorithms, spatial resolution, inter-subject anatomical variability, and partial volume effects between the two coordinate systems. These differences highlight the potential impact of template selection and spatial preprocessing on statistical outcomes, which should be considered when interpreting the present results. Ethnic differences between Chinese and Caucasian populations may affect stereotactic coordinates and atlas-based anatomical localization. As noted in previous work (28), such population variability should be carefully considered when interpreting stereotactic results and performing cross-population comparisons.

Limitations in electrode localization include potential errors from brain shift (due to CSF leakage during surgery) and image fusion artifacts (29). However, postoperative programming can partially compensate for minor electrode misplacement by optimizing stimulation parameters (30). Previous studies reported a positive correlation between GPi-VTA (especially in the sensorimotor region) and BFMDRS improvement. For example, Reich et al. (31) analyzed 87 dystonia patients and found that larger VTA in the GPi sensorimotor region correlated with better motor outcomes. In contrast, our study found no significant correlation between VTA (of GPi or its subregions) and BFMDRS-M improvement. To contextualize our findings, we reference key voxel-based studies that have delineated antidystonic sweet spots and sour spots in dystonia with high spatial resolution (32). In contrast, our regional volume of tissue activated (VTA) analysis summarizes activation across broader GPi subregions, rather than evaluating focal voxel-level effects—this may explain the somewhat weaker correlation between sensorimotor GPi activation and clinical improvement compared with prior voxel-based focal mapping results. We acknowledge that voxel-wise approaches represent an important direction for future work; future studies with standardized imaging and computational workflows could use these methods to refine optimal GPi stimulation targets for isolated generalized dystonia.

Previous studies identified disease duration, baseline motor severity, and genetic factors (e.g., TOR1A mutations) as predictors of DBS outcomes (7). Our observation that higher baseline HAMA scores predicted poorer motor outcomes following GPi-DBS may reflect the highly integrated organization of the cortico-basal ganglia-thalamic (CBGT) network, which interconnects limbic, associative, and motor circuits. In dystonia, anxiety is not merely a comorbid symptom but a marker of more widespread network dysfunction involving both emotional and motor processing (33, 34). Greater preoperative anxiety may thus indicate more extensive network disruption, which could attenuate the therapeutic effect of DBS focused on the sensorimotor GPi. Close interactions between limbic and motor territories within the GPi further support that emotional state can directly modulate motor responses to stimulation (35). Future studies examining the VTA within limbic subregions and longitudinal correlations between affective and motor changes will be necessary to validate these findings.

A key limitation of this study is the relatively small sample size (*n* = 17) compared with the number of variables included in the multivariable linear regression model (8 variables). This may increase the risk of model overfitting, lead to unstable coefficient estimates, and reduce statistical power and generalizability. In addition, owing to the exploratory design and the limited number of *a priori* statistical comparisons, formal correction for multiple testing was not applied. For these reasons, all findings from the regression analyses should be considered preliminary and interpreted with caution. Larger cohorts will be necessary in future studies to validate these results with improved statistical robustness and appropriate multiple comparison adjustments. Single-center, retrospective design: this may introduce selection bias and limit generalizability. Small sample size: especially for the intermediate responder group (*n* = 1), which may affect multi-group comparisons. Lack of STN group analysis: future studies should include STN-DBS patients to compare electrode position/VTA effects across targets.

## Conclusion

Electrode activation contacts closer to the ventral GPi are associated with better BFMDRS-M improvement. No significant correlation exists between BFMDRS-M improvement and VTA of GPi or its subregions. Preoperative anxiety severity is an independent predictor of poor motor outcomes. These findings highlight the importance of precise ventral GPi targeting and preoperative psychological assessment in optimizing DBS outcomes for IGD. Future multi-center, prospective studies with larger cohorts are needed to validate these results.

## Data Availability

The original contributions presented in the study are included in the article/supplementary material, further inquiries can be directed to the corresponding author.
